# Effect of cellulase and lactic acid bacteria on the fermentation quality, carbohydrate conversion, and microbial community of ensiling oat with different moisture contents

**DOI:** 10.3389/fmicb.2022.1013258

**Published:** 2022-10-05

**Authors:** Jinyi Xu, Keyi Zhang, Yufan Lin, Mengxin Li, Xuekai Wang, Qiang Yu, Hong Sun, Qiming Cheng, Yixiao Xie, Chunmei Wang, Ping Li, Chao Chen, Fuyu Yang, Yulong Zheng

**Affiliations:** ^1^College of Animal Science, Guizhou University, Guiyang, China; ^2^College of Grassland Science and Technology, China Agricultural University, Beijing, China

**Keywords:** oat ensiling, moisture content, cellulase, lactic acid bacteria, carbohydrate conversion, microbial community

## Abstract

Oat (*Avena sativa L.*) is one of the most widely cultivated crops used as forage. The aim of this study was to evaluate the effects of cellulase and *Lactobacillus plantarum* interactions with different moisture contents on oat ensiling. Oats with three moisture contents were treated with nothing (C), cellulase (CE), lactic acid bacteria (LP), or CE+LP and ensiled for 30 and 60 days. Compared with the control, LP and CE treatments increased crude protein and lactic acid concentrations and reduced the pH and ammonia nitrogen/total nitrogen (NH_3_-N/TN) ratios of silages. The addition of CE improved lignocellulosic degradation, compared with approximately 67% (LD) and 81% moisture content (HD) ensiling, CE (CE, CE+LP) ensiling in the approximately 75% moisture content (MD) group retained higher water-soluble carbohydrate, glucose, sucrose and fructose concents. The LP and CE inoculations significantly reduced the microbial community diversity, and lower values for the observed species, ACE, Chao1, and Shannon indices compared with CK-treated samples. Additives inhibited the growth of unfavorable bacteria (such as *Clostridium*) and increased the abundances of lactic acid bacteria (LAB); the maximum increases in the *Lactiplantibacillus* abundance were obtained in the LP- and CE+LP-treated samples, improving the microbial community structure in silage. In summary, adding LP and CE effectively improved the oat fermentation quality, and better performances in ensiling oat and lignocellulose degradation were obtained with LP and CE combinations, especially for the MD group of silages that were ensiled for 60 days. The addition of CE and LP at the appropriate moisture content might be helpful for producing high-quality oat silage, and also provide a simple and feasible method to enhance the effects of bacteria and enzymes.

## Introduction

As one of the most cultivated cereals worldwide (Luciano et al., 2022), oat (*Avena sativa L.*) has a high yield and wide adaptability. Oats have earned a global reputation for its high protein content (Zhang et al., 2021), and it has become an important forage for livestock, especially as a supplementary feed during winter (An et al., 2020). In recent years, oat has increased in popularity as important livestock feed (Jia et al., 2020). However, oat is a seasonal crop, the seasonal production of oat seriously hinders its sustainable application in forge rationing, and improperly preserving of oat can waste a large amount of resources. Therefore, developing proper methods to preserve oats is an urgent task.

Ensiling is clearly a practical method for preserving fresh oat and might extend the storage period and year-round supply of oat (Jia et al., 2020). However, as with most forage grasses, oat ensiling involves several factors, which may have individual or interactive effects (such as the moisture content of raw materials, type of microorganisms, and additives used in fermentation) (Zhang et al., 2018). *Lactobacillus plantarum* is considered one of the most versatile lactic acid bacteria (LAB) (McLeod et al., 2019). Many previously published studies have shown that appropriate supplementation with *L. plantarum* enhances fermentation and silage quality, effectively addressing the low numbers of epiphytic LAB in fresh samples (Guo et al., 2020; Mu et al., 2020). However, appropriate moisture is needed for the growth and reproduction of LAB (Troller and Stinson, 1981). In fact, for ensiling, a moisture content that is excessively high tends to promote the growth of *Clostridium* and is detrimental to the growth of LAB (Zheng et al., 2017). Moreover, a lower moisture content may suppress the diffusion of microorganisms, especially LAB, which diffuse more slowly in drier silage than in wet silage (Han et al., 2014). The above studies suggested that an unsuitable moisture content also has a detrimental effect on ensiling.

In addition, oat has a relatively high content of neutral detergent fiber (NDF) and acidic detergent fiber (ADF) (Wang et al., 2020) but is notoriously recalcitrant to degradation (Maruthamuthu et al., 2016). A higher cellulose content is unfavorable for the ensiling process and digestibility of ruminant feed (Ding et al., 2019). Therefore, ensiling with cellulase enzymes might degrade part of the cellulose and provide more water-soluble carbohydrates (WSCs) for the fermentation of LAB (Li M. et al., 2019). However, the interactions of enzymes and bacteria are erratic due to actions on cellulase substrates, which is considered affected by many factors (e.g., cellulase concentration and reaction temperature) (Tan et al., 2018). Li F. H. et al. (2019) found that the effect of the enzyme varies with different moisture contents in raw materials, and pre-treatment with ammonium cellulase exerted better effects on the degradation of lignocellulose in silage with a low dry matter content. All these findings imply that the moisture changes that occur before ensiling could also influence the effect of enzymes on oat ensilage. Therefore, we hypothesized that moisture changes might have different effects on oat ensiling with CE and LAB at different moisture contents.

Thus, our primary study aim was to identify the effects of cellulase and *Lactobacillus* on the quality, transformation of carbohydrates, and microbial community structure of oat silage with different moisture contents as an approach to explore the best treatment methods for oat ensiling.

## Materials and methods

### Raw materials and additives

Milk stage oat was harvested in Zunyi, Guizhou, China, on March 12, 2021, labeled, and returned to the laboratory. The samples were then manually cut into a length of 2–3 cm, divided into three aliquots and processed (by wilting different time and properly replenishing water) to regulate the moisture. During wilting, we use the microwave oven to quickly measure the moisture content of the oats to ensure that the moisture content was regulated as precisely as possible. Finally, we got three different moisture contents (LD: approximately 67%; MD: approximately 75%; HD: approximately 81%). Cellulase and *Lactobacillus* were purchased from Maclean Biological Company. The enzymatic activity of cellulase was 50 U/mg, and the bacterial activity of *Lactobacillus* was 5 × 10^10^ cfu/g.

### Experimental design and preparation of silage

Our 3 × 4 × 2 experiment was performed with a completely randomized design: 3 moisture contents (LD: approximately 67%; MD: approximately 75%; HD: approximately 81%) × 4 additive treatments [no additive control (C); cellulase (CE); lactic acid bacteria (LP); and cellulase+lactic acid bacteria (CE+LP)] × 2 fermentation periods (30 and 60 days). Among them, the inoculation rate of CE was 50 U/g FM, and the inoculation rate of LP was 1 × 10^6^ cfu/g FW. According to the instructions of the agent of LAB, we added the agent into warm water for 2 h to activate it. After ensuring that the lactic acid bacteria were completely activated, the LAB solution and cellulase solution were loaded into pre-prepared sterile and enzyme-free microsprayers, respectively, and then sprayed evenly into each sample to ensure thorough mixing. Three bags of each treatment were prepared. After vacuum sealing, a total of 72 experimental bags were ensiled in an air-conditioned room (25 ± 1°C). The bags were opened after 30 and 60 days of ensiling, then the silages were subsampled for analyses of the chemical composition, fermentation indices, and carbohydrate composition, and the sample at 60 days was also used to analyze the microbial community.

### Sampling and analytical methods

First, to evaluate the fermentation characteristics of silage, 10 g of fresh silage samples from each bag were mixed with 90 ml of sterile superstock water and placed in a refrigerator at 4°C for 24 h. Then, the filtrate was measured immediately with a glass electrode pH meter after the filtrate was repeatedly passed through four layers of medical gauze (filter pore size 11 μm, Biocorp, Nanchang, China) three times. Next, the organic acid concentrations (including lactic, acetic, propionic, and butyric acids) in the silage samples were determined using high-performance liquid chromatography (HPLC) (LC-20A, Shimadzu, Tokyo, Japan) with the method described by Li F. et al. (2019), and the ammonia nitrogen (NH_3_-N) content was measured using a spectrophotometer and the phenol method (Broderick and Kang, 1980). Subsequently, the remaining silage materials were dried in an oven at 65°C for 48 h; then, each sample was immediately weighed to measure the dry matter (DM) content, and the dried samples were separately ground into powders and stored until further use. Finally, the crude protein content was measured using the Kjeldahl method (Association of Official Agricultural Chemists [AOAC], 1995), and the ADF, NDF, cellulose, hemicellulose, and acid detergent lignin (ADL) concents were analyzed using the method reported by Van Soest et al. (1991). Lastly, the WSC, glucose, fructose, and sucrose concents in the silage were quantified using a commercial kit (Beijing solarbio science & technology, Beijing, China).

### Bacterial community analysis

High-throughput sequencing technology can determine millions of DNA molecular sequences at the same time, and the overall microbial community distribution in the silage environment can be comprehensively determined using sequencing (Ron and Liu, 2020). In this assay, DNA was extracted according to the standardized operation of the standard CTAB method. PCR amplifications were conducted with species-specific primers using Barcode, Phusion^®^ High-Fidelity PCR Master Mix with GC Buffer from New England Biolabs, and high fidelity and efficiency enzymes to ensure amplification efficiency and accuracy. The region of amplification was 16Sv34, and the primer sequences were 515 F (CCTAYGGGRBGCAS CAG) and 806R (GGACTACNNGGGTATCTAAT). Finally, we performed several analyses, including species annotation, alpha diversity, beta diversity, and functional prediction, on the acquired valid datasets.

### Data processing and statistical analysis

The data were processed, and statistical analyses were performed using multivariate analysis of variance (ANOVA) of variance with the SPSS program version 19.0 (SPSS Inc. Chicago, IL, USA). Tukey’s multiple comparisons were used to determine differences among means; namely, the effects of cellulase and LAB on fermentation parameters, silage quality, basic nutritional quality, structural carbohydrate contents and non-structural carbohydrate contents in oat silage with different moisture contents were evaluated. *P* < 0.05 was considered a statistically significant difference. All graphical figures and heat-map charts were made with OriginPro 2020.

## Results and discussion

### Characteristics of the fresh material before ensiling

The chemical properties and microbial populations of the fresh oat are shown in Table 1. The crude protein (CP) content of the fresh oat was 83.64 g/kg DM, which was greater than that reported by Wang et al. (2020). However, the WSC content (59.63 g/kg DM) of the fresh oat was lower than that determined by Jia et al. (2020), which may result in a slower growth of LAB due to the lower amount of fermentation substrate at the early stage of ensiling (Ni et al., 2017). In addition, a high content of structural carbohydrates was observed in the fresh oat. The higher fiber content was not conducive to ensiling fermentation and led to a lower feed intake and digestibility in animals (Ding et al., 2019) because microorganisms and enzymes are unable to easily degrade fiber due to its rigid structure (Maruthamuthu et al., 2016). The epibiotic microorganisms on the fresh oats are mainly Enterobacteriaceae and yeasts, and LAB may be unable to become the dominant bacteria due to the lower number of attached LAB (3.96 log cfu/g FM) (He et al., 2021).

**TABLE 1:** Chemical composition and microbial counts of the oats before ensiling.

Items	Fresh oats
pH	6.26 ± 0.003
Dry matter (g/kg FM)	212.00 ± 1.15
Crude protein (g/kg DM)	83.64 ± 3.75
Neutral detergent fiber (g/kg DM)	596.37 ± 23.82
Acid detergent fiber (g/kg DM)	328.63 ± 13.73
Hemicellulose (g/kg DM)	280.82 ± 17.94
Cellulose (g/kg DM)	267.74 ± 19.04
Acid detergent lignin (g/kg DM)	46.30 ± 8.76
Water-soluble carbohydrates (g/kg DM)	59.43 ± 0.46
Glucose (g/kg DM)	23.50 ± 0.61
Fructose (g/kg DM)	15.31 ± 0.13
Sucrose (g/kg DM)	11.13 ± 0.27
Lactic acid bacteria (log cfu/g FM)	3.96 ± 0.14
Enterobacteriaceae (log cfu/g FM)	5.37 ± 0.33
Yeasts (log cfu/g FM)	5.30 ± 0.15

FW, fresh weight; DM, dry matter; SEM, standard error.

### Effects of the enzyme preparation and bacteria on the fermentation characteristics and microbial counts of oat silage with different moisture contents

The fermentation characteristics of oat silage are shown in Table 2. In our study, the interaction of D × T × M existed for NH_3_-N/TN (*P* < 0.001), concentrations of LA (*P* < 0.001), BA (*P* < 0.001), and LA/AA (*P* < 0.001); the interaction of T × M existed for pH (*P* < 0.001), NH_3_-N/TN (*P* < 0.001), concentrations of LA (*P* < 0.001), AA (*P* < 0.001), and LA/AA (*P* < 0.001).

**TABLE 2:** The fermentation characteristics of oat silage at 30 and 60 days.

Items	Moisture	30 Days				60 Days				SEM	Effect (*P*-value)						
		CK	CE	LP	LP+CE	CK	CE	LP	LP+CE		D	T	M	D × T	D × M	T × M	D × T × M
**pH**	LD	5.23^Aa^	4.56^Ba^	4.00^Ca^	3.91^Ca^	5.03^A^	4.49^Ba^	3.83^C^	3.69^C^	0.016	0.036	<0.001	< 0.001	< 0.001	0.105	< 0.001	< 0.01
	MD	4.39^Bb^	4.36^Bb^	3.88^DBb^	3.79^DEb^	4.75^A^	4.11^Cab^	3.74^DE^	3.64^E^								
	HD	4.44^Bb^	3.96^CDb^	4.00^Ca^	3.79^Db^	4.96^A^	3.79^Db^	3.87^CD^	3.59^E^								
**NH_3_-N (g/kg TN)**	LD	84.29^Aa^	61.64^BCa^	34.76^A^	28.01^Da^	68.29^Ba^	59.15^Ca^	29.48^Db^	30.16^Da^	0.381	0.014	<0.001	< 0.001	0.045	< 0.001	< 0.001	< 0.001
	MD	39.09^Bb^	33.63^Cb^	14.84^EFb^	13.08^Fb^	46.53^Ac^	30.19^Dc^	16.78^Ec^	16.69^Eb^								
	MD	42.53^Bb^	34.96^Cb^	23.78^Dc^	15.85^Eb^	54.25^Ab^	38.58^BCb^	33.30^Ca^	26.29^Da^								
**Lactic acid (g/kg DM)**	LD	2.44^Eb^	5.91^Cc^	9.38^Aab^	9.43^Ab^	2.00^Ea^	3.44^Dc^	8.09^Bb^	9.45^Ab^	0.042	< 0.001	<0.001	< 0.001	< 0.001	< 0.001	< 0.001	< 0.001
	MD	5.89^Fa^	7.07^Eb^	10.29^Ba^	13.11^Aa^	1.45^Gb^	5.45^Fb^	7.98^Db^	9.26^Cb^								
	HD	5.49^Da^	11.08^Ba^	9.29^Cb^	12.95^Aa^	0.90^Ec^	10.84^Ba^	9.51^Ca^	13.51^Aa^								
**Acetic acid (g/kg DM)**	LD	1.33^Aa^	1.02^BC^	1.08^AB^	0.92^BCb^	0.74^CDa^	0.58^Dab^	0.83^BCD^	0.85^BCD^	0.017	< 0.001	<0.001	< 0.01	0.095	< 0.01	< 0.001	0.250
	MD	0.58^Cb^	0.89^B^	1.16^A^	1.18^Aa^	0.26^Db^	0.40^Db^	0.88^B^	0.83^B^								
	HD	0.46^Db^	0.8^BC^	1.09^AB^	1.13^Aa^	0.43^Db^	0.69^CDa^	0.96^ABC^	1.16^A^								
**Lactic acid/acetic acid**	LD	1.84^Cb^	5.91^Bc^	8.72^AB^	10.27^A^	2.74^Cb^	6.13^Bb^	9.83^A^	12.06^A^	0.217	0.886	<0.001	< 0.001	< 0.001	0.098	< 0.001	< 0.001
	MD	10.64^BCa^	7.9^CDb^	8.91*^B^*C	11.11^AB^	5.8^CDa^	13.69^Aa^	9.11^BC^	11.68^AB^								
	HD	12.00^BCa^	13.79^ABa^	8.54^C^	11.64^BC^	2.09^bD^	16.24^Aa^	10.97^BC^	11.67^BC^								
**Propionic acid (g/kg DM)**	LD	1.17^AB^	1.17^AB^	1.23^AB^	1.26^AB^	1.2^ABa^	0.93^Bb^	1.43^Aab^	1.43^A^	0.021	0.086	<0.001	< 0.001	< 0.001	< 0.001	0.103	0.244
	MD	1.07^A^	1.10^A^	1.15^A^	1.10^A^	0.29^Bb^	0.50^Bc^	1.04^Ab^	1.07^A^								
	HD	1.11^BC^	1.17^ABC^	1.18^ABC^	1.12^BC^	0.96^Ca^	1.31^ABa^	1.47^Aa^	1.32^AB^								
**Butyric acid (g/kg DM)**	LD	0.11^Bb^	0.08^Bb^	0.28^B^	0.22^Bb^	1.79^Ab^	1.71^A^	0.20^B^	0.18^B^	0.055	< 0.001	<0.001	0.048	< 0.01	0.873	< 0.01	< 0.001
	MD	0.79^BCab^	0.67^BCa^	0.26^A^	0.34^Aa^	3.08^A^	1.64^B^	0.17^A^	0.15^A^								
	HD	1.11^Ba^	0.56^Bab^	0.16^B^	0.27^Bab^	3.62^A^	0.60^B^	0.26^B^	0.25^B^								

CK, control; LP, *Lactobacillus plantarum*; CE, cellulase; LP+CE, combination of *Lactobacillus plantarum* and cellulase. Different lowercase letters show significant differences among the same additive treatments in the different moisture contents (*P* < 0.05); different capital letters show the same significant differences among moisture contents in the different additions (*P* < 0.05). DM, dry matter; NH_3_-N, ammonia nitrogen; TN, total nitrogen; LD, the moisture content is approximately 67%; MD, the moisture content is approximately 75%; LD, the moisture content is approximately 81%; SEM, standard error of means; D, ensiling days; T, additive; M, moisture content; D × T, the interaction between the additive and ensiling days; D × M, the interaction between ensiling days and the moisture content; T × M, the interaction between the additive and moisture content; and D × T × M, the interaction between ensiling days, the additive and moisture content.

The pH of silage is an important parameter to evaluate the quality of the ensiling effect (Ren et al., 2021), and the quality of silage can only be guaranteed when the pH is less than 4.2. In this study, the pH of the additive treatment samples was lower than that of the CK treatment samples. The addition of LAB and CE both significantly accelerated the decrease in pH (*P* < 0.01), and the lower pH effectively inhibited the growth and enzymatic activity of undesirable microorganisms (Kung et al., 2018). However, compared with the CE-treated samples, the LP-treated samples exhibited lower pH values (<4.2), which might be mainly due to the differences in the mechanisms of action between CE and LP; compared to CE additions, LP additions often lead to more rapid fermentations (Li et al., 2018). In fact, as seen from Table 2, the moisture content also caused significant effects on the pH (*P* < 0.01). Overall, the pH of the MD group was significantly lower than that of the LD and HD groupsat 30 days of fermentation, probably because LAB produced more organic acids in the MD group, as evidenced by the lactic acid content. The moisture content in the MD group might be more suitable for the metabolism of LAB, thus promoting increased production of lactic acid (Troller and Stinson, 1981).

In silage, higher NH_3_-N levels are indicative of excessive protein degradation (Mu et al., 2020), and in this study, the NH_3_-N/TN content was less than 10% in all groups. As expected, the NH_3_-N/TN was significantly higher in the control group than in the other groups. Similar to the effect of pH, the NH_3_-N/TN value of the LP-added treatment samples was significantly lower than that of the CE-added treatment samples (*P* < 0.05). The reason for the decreased NH_3_-N content was probably because inoculation with LAB reduced the growth of *Clostridium* and the trypsin activity of mycobacteria (Oliveira et al., 2017), as shown in Figure 3B; the relative abundance of *Clostridium_sensu_stricto_12* in CK was higher than that in the other treatment groups. Moreover, for the same treatment with different moisture contents, the NH_3_-N/TN content in the MD group was lower than that in the HD and LD groups, especially in the CE+LP treatment samples, so more crude protein was preserved (Table 5).

**FIGURE 1:**
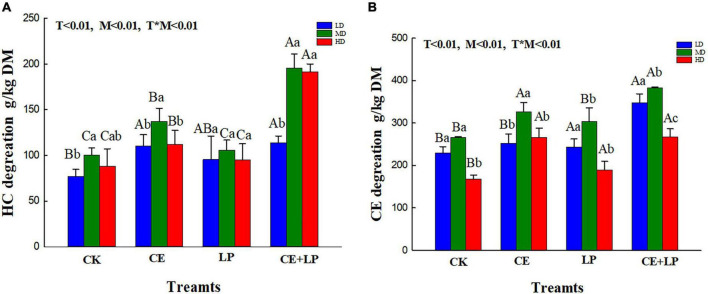
Effects of additives and the moisture content on the hemicellulose degradation rate **(A)** and cellulose degradation rate **(B)** in the oats at 60 days of silage. CK, control; LP, *L. plantarum*; CE, cellulase; LP+CE, combination of *L. plantarum* and cellulase. Different lowercase letters show significant differences among the same additive treatments in the different moisture contents (*P* < 0.05). Different capital letters indicate the same significant differences among moisture contents in the different additive-treated groups (*P* < 0.05). DM, dry matter; TN, total nitrogen; LD, the moisture content is approximately 67%; MD, the moisture content is approximately 75%; HD, the moisture content is approximately 81%; SEM, standard error of means; T, additive; M, moisture content; T × M, the interaction between the additive and moisture content.

**FIGURE 2:**
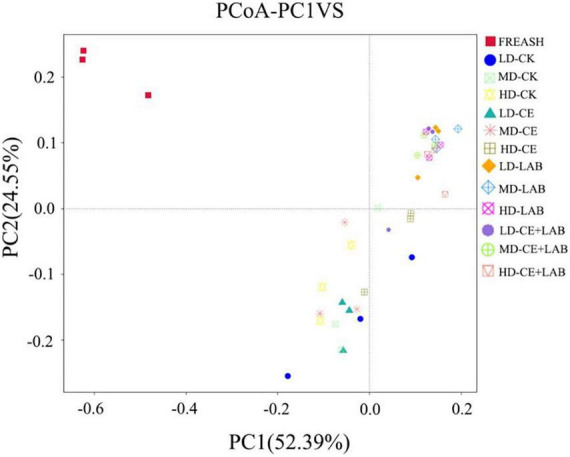
Principal coordinate analysis (PCoA) of oat silage on day 60. FRESH, fresh oat sample; LD, the moisture content is approximately 67%; MD, the moisture content is approximately 75%; HD, the moisture content is approximately 81%; CK, control, no additive; LP, *L. plantarum*; CE, cellulase; LP+CE, combination of *L. plantarum* and cellulase.

**FIGURE 3:**
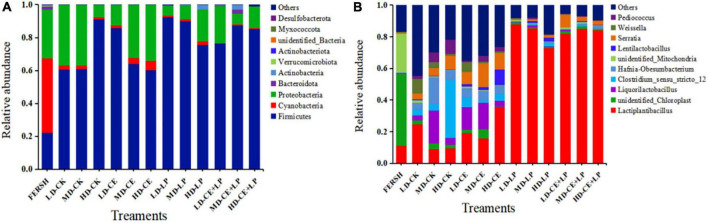
The relative abundance of bacteria based on the taxonomic classification of the microbial community of oat silage on day 60 identified at **(A)** the phylum level and genus level **(B)**. FRESH, fresh sample; LD, the moisture content is approximately 67%; MD, the moisture content is approximately 75%; HD, the moisture content is approximately 81%; CK, control, no additive; LP, *L. plantarum*; CE, cellulase; LP+CE, combination of *L. plantarum* and cellulase.

**TABLE 3:** The microbial counts of oat silage at 30 and 60 days.

Items	Moisture	30 Days				60 Days				SEM	Effect (*P*-value)						
		CK	CE	LP	LP+CE	CK	CE	LP	LP+CE		D	T	M	D × T	D × M	T × M	D × T × M
**Lactic acid bacteria log cfu/g FM**	LD	6.90^AB^	7.18^AB^	6.15^BCb^	6.59^AB^	7.67^A^	6.87^ABa^	5.98^BC^	5.56^Cb^	0.055	0.081	<0.001	<0.001	0.014	0.063	0.027	0.151
	MD	7.19^AB^	7.22^AB^	6.90^Ba^	6.89^B^	7.80^A^	7.09^ABa^	7.13^AB^	6.76^Ba^								
	HD	6.96^AB^	7.23^A^	6.80^ABCa^	6.42^CD^	6.44^BCD^	6.08^Db^	6.90^ABC^	5.98^Dab^								
**Enterobacteriaceae log cfu/g FM**	LD	0.837^A^	0.00^B^	0.00^B^	0.00^B^	0.00^B^	0.00^Bb^	0.241^B^	0.256^B^	0.028	0.037	<0.001	<0.001	<0.001	<0.001	<0.001	<0.001
	MD	0.00	0.00	0.00	0.00	0.00	0.285^ab^	0.30	0.55								
	HD	0.00^B^	0.00^B^	0.00^B^	0.03^B^	0.27^B^	0.78^Aa^	0.00^B^	0.00^B^								
**Yeasts log cfu/g FM**	LD	3.924^Aa^	0.00^B^	0.00^A^	3.35B	0.90^B^	0.00^B^	0.00^B^	1.30^B^	0.205	0.131	<0.01	0.550	0.134	0.008	0.293	0.815
	MD	1.133^B^	0.00	0.00	2.06	1.13	3.03	1.96	3.48								
	HD	0.00^Bb^	0.00B	0.00^B^	1.20^AB^	1.18^AB^	3.39^A^	0.00^B^	2.84^B^								
**Molds log cfu/g FM**	LD	0.00	0.00	0.00	0.00	0.00	0.00	0.00	0.00	ND	ND	ND	ND	ND	ND	ND	ND
	MD	0.00	0.00	0.00	0.00	0.00	0.00	0.00	0.00								
	HD	0.00	0.00	0.00	0.00	0.00	0.00	0.00	0.00								

CK, control; LP, *Lactobacillus plantarum*; CE, cellulase; LP+CE, combination of *Lactobacillus plantarum* and cellulase. Different lowercase letters show significant differences among the same additive treatments in the different moisture contents (*P* < 0.05); different capital letters show the same significant differences among moisture contents in the different additions (*P* < 0.05). DM, dry matter; NH_3_-N, ammonia nitrogen; TN, total nitrogen; ND, none Detected; LD, the moisture content is approximately 67%; MD, the moisture content is approximately 75%; LD, the moisture content is approximately 81%; SEM, standard error of means; D, ensiling days; T, additive; M, moisture content; D × T, the interaction between the additive and ensiling days; D × M, the interaction between ensiling days and the moisture content; T × M, the interaction between the additive and moisture content; D × T × M, the interaction between ensiling days, the additive and moisture content.

**TABLE 4:** Effects of additives and moisture content on structural carbohydrate concents.

Items	Moisture	30 Days				60 Days				SEM	Effect (*P*-value)						
		CK	CE	LP	LP+CE	CK	CE	LP	LP+CE		D	T	M	D × T	D × M	T × M	D × T × M
**NDF g/kg DM**	LD	491.6Ab	461.08^Ca^	486.8^AB^	429.3^Eb^	490.03^Ab^	476.42^Ba^	481.67^ABab^	442.82^Da^	1.072	0.239	<0.001	<0.001	<0.001	0.208	<0.001	0.253
	MD	498.75Ab	428.74^Eb^	486.1^AB^	404.88^Fa^	474.45^Cc^	447.34^Db^	462.11^Db^	420.38^Fb^								
	HD	510.40Aa	461.03^Ca^	497.51^A^	433.09^Da^	506.56^Aa^	471.42^Bab^	494.56^Aa^	449.57^Da^								
**ADF g/kg DM**	LD	287.62A	270.55^Ba^	282.46^A^	247.62^C^	242.98^Cb^	238.21^Ca^	239.54^Cab^	205.53^Db^	1.092	<0.001	<0.001	<0.001	<0.001	<0.001	<0.001	0.204
	MD	288.63A	241.58^Bb^	285.77^A^	228.01^BC^	233.55^BCb^	216.33^CDb^	222.63^BCDb^	204.91^Db^								
	HD	280.40A	241.34^Cb^	280.29^A^	235.72^CD^	262.4^Ba^	233.74^Da^	252.21^BCa^	233.11^Da^								
**Hemicellulose g/kg DM**	LD	203.98Cb	190.53^Db^	204.35^C^	181.68^E^	247.05^A^	238.21^AB^	242.13^AB^	237.28^Ba^	1.187	<0.001	<0.001	<0.001	0.552	<0.001	0.552	0.837
	MD	210.12Cb	187.16^Eb^	200.33^D^	176.87^E^	240.91^A^	231.01^AB^	239.47^A^	215.47^Cb^								
	HD	230ABa	219.69^ABa^	217.21^BC^	197.36^C^	244.16^A^	237.68^AB^	242.35^A^	216.46^BCa^								
**Cellulose g/kg DM**	LD	256.03A	242.05^Ba^	254.08^A^	217.79^C^	216.41^Cab^	209.89^Ca^	212.49^Cab^	183.17^Db^	0.940	<0.001	<0.001	<0.001	<0.001	<0.001	<0.001	0.546
	MD	253.77A	213.11^Bb^	252.54^A^	201.26^BC^	206.12^BCb^	188.99^CDb^	195.6^BCb^	173.38^Db^								
	HD	253.81A	213.67^CDb^	254.24^A^	209.32^D^	233.79^Ba^	206.06^Da^	227.62^BCa^	205.72^Da^								
**ADL g/kg DM**	LD	26.25	25.31	22.86	24.72	24.11	23.72	24.13	20.37	0.528	0.384	0.909	0.188	0.646	0.873	0.649	0.296
	MD	28.73	24.07	29.66	22.15	24.39	24.70	24.06	29.02								
	HD	23.14	24.43	23.54	24.35	24.17	23.78	20.51	25.14								
**AIA g/kg DM**	LD	5.35	3.19	5.52^A^	5.11	2.46	4.60	2.93	1.99	0.235	0.051	0.474	0.506	0.467	0.033	0.467	0.652
	MD	6.13	4.41	3.57^ab^	4.59	3.04	2.64	2.97	2.50								
	HD	3.45	3.25	2.51^b^	2.05	4.44	3.90	4.09	2.25								

CK, control; LP, *Lactobacillus plantarum*; CE, cellulase; LP+CE, combination of *Lactobacillus plantarum* and cellulase. Different lowercase letters show significant differences among the same additive treatments in the different moisture contents (*P* < 0.05), different capital letters show the same significant differences among moisture contents in the different additions (*P* < 0.05). DM, dry matter; TN, total nitrogen; LD, the moisture content is approximately 67%; MD, the moisture content is approximately 75%; LD, the moisture content is approximately 81%; NDF, neutral detergent fiber; ADF, acid detergent fiber; ADL, acid detergent lignin; AIA, acid-insoluble ash; SEM, standard error of the mean; D, ensiling days; T, additive; M, moisture content; D × T, the interaction between the additive and ensiling days; D × M, the interaction between ensiling days and the moisture content; T × M, the interaction between the additive and moisture content; and D × T × M, the interaction between ensiling days, the additive and moisture content.

**TABLE 5:** Effects of the additives and moisture content on the basic nutritional quality and non-structural sugar composition of the oat silage.

Items	Moisture	30 Days				60 Days				SEM	Effect (*P*-value)						
		CK	CE	LP	LP+CE	CK	CE	LP	LP+CE		D	T	M	D × T	D × M	T × M	D × T × M
**DM g/kg FM**	LD	255.77^c^	260.10^a^	258.16^a^	264.68^a^	252.80^c^	254.21	257.05^a^	260.59^a^	1.354	<0.001	<0.001	<0.001	<0.001	<0.001	0.037	0.131
	MD	199.02^Cb^	204.72^BCb^	213.63^ABb^	221.00^Ab^	197.54^Cb^	201.57^C^	211.67^ABb^	245.05^ABb^								
	HD	184.52B^Ca^	188.04^ABCc^	187.07^ABCc^	194.99^Ac^	181.80^Ca^	185.21^BC^	183.59^BCc^	191.41^ABc^								
**CP g/kg DM**	LD	73.12^b^	76.00^b^	76.40	78.183^b^	75.93	78.62	79.51^a^	78.86	0.782	0.495	<0.01	<0.001	0.618	0.018	5.543	0.711
	MD	83.43^Ba^	84.58^Ba^	83.95^B^	94.13^Aa^	76.03^CD^	80.56^BC^	80.12^BCa^	85.12^B^								
	HD	71.35^b^	74.36^b^	76.10	82.63^b^	77.32	79.10	70.76^b^	81.98								
**WSC g/kg DM**	LD	21.59^CDb^	25.47^B^	25.12^BC^	30.81^A^	21.07^D^	21.83^CDb^	22.76^BCD^	24.67^BCDb^	0.163	<0.001	<0.001	<0.01	0.180	0.692	0.661	0.145
	MD	23.94^Ca^	26.85^B^	25.12^BC^	30.07^A^	20.63^D^	24.10^Ca^	23.37^C^	27.77^Ba^								
	HD	22.56^Dab^	26.2^B^	24.33^CD^	28.11^A^	20.56^E^	22.81^Dab^	22.70^D^	25.56^Dab^								
**Glucose g/kg DM**	LD	2.39^CDc^	3.82^Bb^	2.98^C^	4.68^Ab^	1.85^Db^	2.57^CDb^	2.46^CDb^	4.46^AB^	0.047	<0.001	<0.001	<0.001	<0.01	<0.001	<0.001	<0.001
	MD	3.80^CDa^	5.92^Aa^	3.09^D^	6.17^Aa^	2.21^Ea^	4.66^Ba^	3.26^Da^	4.30^BC^								
	HD	3.07^Cb^	4.13^Bb^	2.39^D^	3.64^Bb^	2.10^Da^	4.11^Ba^	2.11^Db^	4.74^A^								
**Fructose g/kg DM**	LD	8.73^BCab^	11.93^AB^	12.44^ABa^	13.80^A^	5.96^Cb^	9.15^BC^	8.38^BC^	11.69^AB^	0.365	0.029	<0.001	0.003	0.922	0.112	0.762	0.799
	MD	12.65^ABa^	14.70^AB^	13.05^ABa^	17.64^A^	9.86^Ba^	12.99^AB^	10.28^B^	14.59								
	HD	5.91^Bb^	13.56^A^	7.35^ABb^	14.07^A^	8.18^ABa^	11.93^AB^	10.57^AB^	12.43^AB^								
**Sucrose g/kg DM**	LD	5.05^C^	8.98^A^	5.44^Cb^	9.50^Aa^	2.62^D^	5.07^C^	6.78^Ba^	7.21^B^	0.202	<0.001	<0.001	0.596	0.528	0.223	0.037	<0.01
	MD	4.99^CD^	5.15^CD^	9.43^Aa^	5.48^BCDb^	4.60^CD^	6.05^BC^	4.20^Db^	6.85^B^								
	HD	5.75^AB^	8.04^AB^	6.65^ABb^	9.48^Aa^	3.74^B^	5.6^AB^	2.65^Bc^	7.62^AB^								

CK, control; LP, *Lactobacillus plantarum*; CE, cellulase; LP+CE, combination of *Lactobacillus plantarum* and cellulase. Different lowercase letters show significant differences among the same additive treatments in the difference in moisture content (*P* < 0.05). Different capital letters show the same significant differences among moisture contents in the difference additive (*P* < 0.05). DM, dry matter; FW, fresh weight; LD, the moisture content is approximately 67%; MD, the moisture content is approximately 75%; LD, the moisture content is approximately 81%; CP, crude protein; WSC, water-soluble carbohydrate; SEM, standard error of the mean; D, ensiling days; T, additive; M, moisture contents; D × T, the interaction between the additive and ensiling days; D × M, the interaction between ensiling days and the moisture content; T × M, the interaction between the additive and moisture content; D × T × M, the interaction between ensiling days, the additive and moisture content.

In addition, CE and LP supplementation exerted significant effects on the concentrations of lactic acid (LA), acetic acid (AA), propionic acid (PA), and butyric acid (BA) in oat silage (*P* < 0.05). The lactic acid is among the important substances that decreases the silage pH and improves the quality of fermentation (Ni et al., 2017). The LP treatment significantly increased the LA concentration for the three moisture levels of the silages, and this result could be attributed to the inoculation of homofermentative LAB (LP), which can efficiently transform WSC into LA (Mu et al., 2020). The LA content in the LP+CE treatment samples was significantly higher than that in the LP treatment samples, which confirmed a change in the silage pH. The LA concentration of additive-treated samples (CE, LP, CE+LP) in the HD group was higher than that in the LD and MD groups after ensiling for 60 days, and this phenomenon may be due to the lower DM content in the HD group of silage, and this phenomenon may be due to the lower DM content in the HD group of silage. However, for lactic acid/acetic acid (LA/AA) content, CE and LP additions both improved homofermentation, which was analogous to the findings of Su et al. (2019). Furthermore, previous studies found that PA and BA are undesirable for silage (Dong et al., 2020), since their production often leads to a significant amount of energy loss. In our study, propionic acid and butyric acid concentrations were low in all treatment groups. Butyric acid is generally produced by *Clostridium* (Wang et al., 2019); therefore, the decreased concentration of butyric acid may be due to weakened growth of *Clostridium*, which can also be found from the lower abundance of *Clostridium* in the additive treatment samples (Figure 3).

The microbial counts of oat silage are listed in Table 3. Interestingly, compared to the CK-treated samples (*P* < 0.05), the LP- and CE-treated samples exhibited fewer quantities of LAB; the reason for this phenomenon may be the lower pH at the late stage of ensiling, and many LAB strains are less tolerant to the lower pH value (Ohmomo et al., 2002). However, for the quantity of Enterobacteriaceae, the silage treatment was less than 1.00 log10 cfu⋅g^–1^ FM, while the yeast quantity was less detected at 30 days, and no molds were detected in any of the treatment samples at 30 and 60 days. The ANOVA results showed that moisture content treatment had an extremely significant effect on the number of LAB and yeast (*P* < 0.01), and additive treatment had an extremely significant effect on the LAB and Enterobacteriaceae quantities in the silages (*P* < 0.001).

### Effects of additives and moisture on structural carbohydrate contents in the ensiling of oats

The impacts of additives and moisture on the structural carbohydrate composition of oat silage [mainly NDF, ADF, hemicellulose, cellulose, ADL, and acid insoluble ash (AIA)] are summarized in Table 4. The additives and moisture significantly influenced the NDF, ADF, hemicellulose and cellulose contents in all treated silages (*T* < 0.05, *M* < 0.05), but not the ADL and AIA contents (*T* > 0.05, *M* > 0.05), and the interaction of T × M existed for concerns of NDF (*P* < 0.001), ADF (*P* < 0.001), hemicellulose (*P* < 0.001), cellulose (*P* < 0.001).

Previous studies showed that ensiling fermentation with cellulase effectively decreases the NDF and ADF concents (Su et al., 2019). In our study, the CE addition hydrolyzed lignocellulose, resulting in significantly reduced NDF, ADF, hemicellulose and cellulose concents in the silages with the three moisture levels (*P* < 0.05). The low pH of LAB-treated silage and high LA concentration may have caused the decreased lignification of the structural carbohydrates, perhaps increasing the digestibility of the silage (Kaewpila et al., 2021).

Interestingly, for the CE addition alone, we found that the cellulose and hemicellulose degradation rate of the CE treatment samples in the MD groups was better than that in the HD group and LD (Figure 1), and the effect differed with moisture content, which may be attributable to the knowledge that cellulase degradation efficiency is often affected by numerous factors (e.g., the substrate concentration, enzyme concentration, and reaction conditions) (Zhou et al., 2018). Specifically, the enzymatic hydrolysis of plants mainly involves a process of transfer from the liquid medium (enzyme solution) to the surface of the solid substrate (plant cellulose), which is primarily governed by diffusion movements. Hence, the diffusion rate of the enzyme macromolecules determines the overall reaction rate of enzymatic hydrolysis (Yachmenev et al., 2009). In the current study, although the high moisture content increased the diffusion rate of enzyme molecules to some extent, the moisture content also caused the enzyme solution to be excessively diluted and decreased the enzyme activity. Conversely, a forage moisture content that is too low may decrease the transport medium in the system, thereby leading to a lower rate of diffusion and reducing the overall rate of hydrolysis. Thus, our research revealed that the appropriate water content of the MD group promoted enzymatic hydrolysis of oat silages, leading to a greater release of soluble sugars.

As expected, the combined addition of LP and CE resulted in better degradation of lignocellulose compared to that of the addition of CE alone, and lower NDF, ADF, hemicellulose and cellulose concents (*P* < 0.05) were detected in CE+LP-treated silages, which might be attributed to the synergistic effect with LAB and CE (Li F. H. et al., 2019). According to previous research, CE may effectively degrade cellulose into monosaccharides, which might increase the amount of fermentation substrate for LAB (So et al., 2022). Additionally, the intake of monosaccharides fosters enzymatic hydrolysis (Li F. H. et al., 2019). Moreover, treatment with CE+LP resulted in the lowest ADF, NDF, hemicellulose, and hemicellulose contents in the MD groups after ensiling for 60 days. This result may be because the moisture content of the MD group was suitable for cellulase; the pH is also among the important factors that affect the activity of enzymes (Tay et al., 2016). Regarding the metabolic reaction of LAB, the moisture content of MD might be more suitable in the current study (Troller and Stinson, 1981). The rapid growth of LAB in the MD group could quickly decrease the pH below 5.5, which might contribute to cellulase exhibiting the best enzymatic activity earlier (based on the manufacturer’s instructions, the optimum pH of the cellulase action environment is approximately 4–5.5). The above analysis shows that a suitable moisture content can further enhance the collaborative effect of enzymes and bacteria.

Moreover, for ADL and AIA, all the treatments had no significant difference compared with that of CK (*T* > 0.05, *M* > 0.05). The potential explanation is that lignin is a three-dimensional polymer molecule composed of three different phenyl propane precursor monomer units (Maurya et al., 2015), and ADL and AIA are the most non-biodegradable components in the plant cell wall. Therefore, further research is still needed.

### Effects of enzyme and bacterial additives on the conversion of non-structural sugars in silage with different moisture contents *in vitro*

The effects of different additives and moisture contents on the DM, CP, WSC, sucrose, fructose and glucose concents in oat silage are shown in Table 5. In our study, the interaction of D × T × M existed for concents of glouse (*P* < 0.001). The DM was better preserved in all groups after ensiling for 30 and 60 days, and the DM in the samples treated with additives (LP, CE, LP+CE) in the three moisture groups was significantly higher than that in the CK-treated samples (*P* < 0.01). This result might be due to a decrease in pH caused by LAB and CE additions; the lower pH decreased the growth of most bacteria and thus preserved more DM (Dong et al., 2018). As shown in Table 5, the additives also exerted significant effects on CP contents (*P* < 0.01) and generated a higher CP content than that in the CK-treated samples, consistent with the findings reported by Mu et al. (2020). The above result may be due to the relatively low pH value, which could limit protease activity and microbial breakdown (Gong and Qi, 2020). Moreover, the effect of the moisture content on the CP content was significant (*P* < 0.01), as CP was better preserved in the MD-CE+LP group of silages (85.12 g/kg DM) than in the LD and HD groups. The best protein preservation was observed in the MD group, consistent with the NH_3_-N content. The result may be due to the large amount of unfavorable bacterial reproduction caused by wilting in the LD group, while the result obtained for the HD group could be due to poor fermentation.

We also measured the concents of non-structural carbohydrates (including WSC, glucose, fructose, and sucrose) in silages to better assess the effects of the additives and moisture content on the degradation of structural carbohydrates (Table 5). ANOVA showed that additives, moisture and their synergy had significant effects on WSC, glucose, and fructose (*P* < 0.01). Table 5 revealed that the non-structural carbohydrates in silage on day 60 were overall lower than those on day 30 and decreased with ensiling time. This is a common phenomenon due to the consumption of substrate by microorganisms during fermentation (Li F. H. et al., 2019). Compared with CK, the WSC concents were significantly increased in the additive-treated samples (CE, LP, CE+LP), especially in the CE+LP-treated samples. The increase in the WSC content in the CE-treated samples was mainly due to the degradation of fiber, whereas in the LP-treated samples, the increase may be influenced by the increased acidification due to the lower pH, which might preserve more WSC (Mu et al., 2020). The WSC content in the CE- and LP-treated samples was slightly lower than that of the CE+LP-treated samples, which further demonstrated the synergistic effect between LAB and CE. Moreover, our results showed that before and after ensiling, glucose was the most consumed carbohydrate over fructose and sucrose. Tao et al. (2003) showed that glucose is the preferred substrate for LAB, consistent with our findings. The glucose, fructose and sucrose concents were lower in the LP-treated samples than in the CE-treated samples from all moisture content groups after day 60, indicating that cellulase is more effective at increasing the production of non-structural sugars than LAB.

Unexpectedly, compared to the LD, MD and HD groups, the silage treated with CE (CE, LP+CE) contained different glucose concents, with the highest content detected in the MD group. Glucose is the final hydrolysis product of cellulose degradation by cellulase (Xu and Ding, 2007), which, to some extent, reflects the high efficiency of the enzymatic reaction. According to the classical enzyme-catalyzed reaction mode, the following relationship is obtained:


$$E+S\,\begin{array}{c} \overset{k\,1}{⟶}\\ \overset{\leftarrow }{k\,2} \end{array}\,\left[\mathrm{ES}\right]\overset{k\,3}{⟶}E+P$$

where E represents the enzyme; S is the substrate; ES is the enzyme-substrate complex; P is the product; k1 and k2 are the rate constants for adsorption and desorption, respectively; and k3 is the rate constant for product formation (Savageau, 1995). The changes in K1, K2, and K3 directly affect the efficiency of the enzyme-catalyzed reaction. In this mechanism, the rate of product generation determines the rate of the whole enzyme-catalyzed reaction. In the present study, a low moisture content easily resulted in a smaller K1, while a high moisture content increased K2, and the changes in K1 and K2 decreased K3. This caused the enzymes in the MD group to exhibit a higher catalytic efficiency than that in the LD and HD groups. Similar to glucose, for most silage samples, the WSC and fructose in the MD group were higher than those in the LD and HD groups (*P* < 0.05). Therefore, treatment of the MD group of silages with the additives exerted a better effect on preserving the non-structural carbohydrates.

### Effects of enzyme and bacterial additives on the bacterial community in oat silage with different moisture contents

As displayed in Table 6, the alpha diversity indices of fresh samples and oat after 60 days of ensiling were determined using 16S RNA sequencing. The highest observed species, ACE, Chao1, and Shannon values in fresh samples were observed, and a significant decrease in bacterial diversity occurred in all silages after 60 days of fermentation. The values for the observed species, ACE, Chao1, and Shannon indices of the LP- and LP+CE-treated samples were lower than those for the CK-treated samples, consistent with a previous study by He et al. (2021). Meanwhile, the principal coordinate analysis (PCoA) of the bacterial community is shown in Figure 2. The CK-treated samples were mainly clustered in the third quadrants, whereas most of the CE inoculated silages were clustered together in the third and fourth quadrants. However, the samples treated with LP (the LP and LP+CE treatment groups) were basically clustered in the first quadrant. Based on these results, LP and CE inoculation significantly altered the bacterial community structure in silage.

**TABLE 6:** Diversity and richness of the bacterial microbiota of oat silage.

Sample name	Observed species	ACE	Chao1	Shannon	Goods coverage
**Fresh**	360^A^	418.04^A^	387.95^A^	4.15^A^	0.999^A^
**LD-CK**	178^E^	194.23^F^	187.23^F^	3.63^D^	0.999^A^
**MD-CK**	214^C^	287.49^B^	268.4^B^	2.23^G^	0.999^A^
**HD-CK**	154^H^	187.43^G^	182.63^G^	3.49^E^	0.999^A^
**LD-CE**	164^F^	178.51^H^	177.45^H^	3.76^C^	0.999^A^
**MD-CE**	204^D^	250.02^D^	246.09^D^	4.04^B^	0.999^A^
**HD-CE**	163^F^	187.34^G^	182.03^G^	2.73^F^	0.998^A^
**LD-LP**	140^J^	168.1^I^	153.24^J^	0.85^K^	0.999^A^
**MD-LP**	146^I^	178.62^H^	162.25^I^	1.25^I^	0.998^A^
**HD-LP**	133^K^	145.57^K^	140.96^L^	2.15^H^	0.999^A^
**LD-CE+LP**	157^G^	214.73^E^	197.62^E^	0.96^J^	0.999^A^
**MD-CE+LP**	229^B^	267.86^C^	251.72^C^	1.21^I^	0.999^A^
**HD-CE+LP**	124^L^	156.22^J^	145.88^K^	0.95^J^	0.998^A^
**SEM**	0.66	0.27	0.44	0.01	0.00
***P*-value**	<0.001	<0.001	<0.001	<0.001	0.648

CK, control; LP, *L. plantarum*; CE, cellulase; LP+CE, combination of *L. plantarum* and cellulase. Fresh, fresh oat sample; LD, the moisture content is approximately 67%; MD, the moisture content is approximately 75%; LD, the moisture content is approximately 81%. SEM, standard error of means. Significant differences (*P* < 0.05) among the different treatments are indicated by capital letters.

The relative abundance of bacterial communities at the phylum level on fresh oat samples and after 60 days of silage are shown in Figure 3A. The most abundant phyla in the fresh oat samples were mainly *Cyanobacteria*, *Proteobacteria*, and *Firmicutes*. After 60 days of ensiling, the abundance of the *Cyanobacteria* phylum was greatly reduced, and the *Firmicutes* phylum increased to become the new dominant phylum. The addition of LAB and CE contributed to the increase in the abundance of the *Firmicutes* phylum in the CE+LP silage samples, consistent with the findings reported by Ren et al. (2020). The CE+LP-treated samples in the MD group exhibited a higher abundance of the *Firmicutes* phylum than the LD and HD groups, which might be because the addition of CE at the appropriate moisture content (MD) produced more fermentation substrates, improving the growth of LAB (Li F. H. et al., 2019). Bacteria in the phylum *Firmicutes* produce acid (Zhao et al., 2017) and rapidly decrease the pH of silage (Table 2), inhibiting the growth of undesirable microorganisms. The *Proteobacteria* phylum plays a pivotal role in fermentation, but the *Proteobacteria* phylum includes various pathogenic genera (Du et al., 2021). In our study, an overall trend toward a decrease in the relative abundance of the *Proteobacteria* phylum was observed at the end of ensiling (except for the LD-CK, LD-CE, and MD-CE treatment samples), especially for the LP- and LP+CE-treated samples in the MD groups. This result may be attributed to the observation that the acidic and anaerobic environment in the MD groups is more conducive to the proliferation of *Firmicutes* bacteria (Keshri et al., 2018).

Figure 3B shows the relative abundance of the major bacteria at the genus level before and after oat ensiling. The fresh samples contained a low abundance of *Lactiplantibacillus*, indicating that not many LAB were attached in the fresh materials, as seen in Table 1. Compared to the CK- and CE-treated samples, the maximum increases in the *Lactiplantibacillus* abundance were obtained with the LP- and CE+LP-treated samples, reaching more than 75% and becoming the most dominant genus. *Lactobacilli* are important in the accumulation of LA and decline in pH (Wang et al., 2020), and their higher abundance often results in a better quality of fermentation. In contrast, the CK- and CE-treated samples showed a small increase in *Pediococcus* abundance, and small quantities of *Weissella* were also detected. *Weissella* is a heterotypic fermentation LAB that can produce LA and AA (Graf et al., 2016) and is an important LAB that is present in high-quality silage (Ni et al., 2017). For the different moisture contents in the same treatment, the lowest abundance of LAB (mainly *Lactiplantibacillus*, *Liquorilactobacillus*, *Lentilactobacillus*, *Pediococcus*, and *Weissella*) was observed in the HD group with the LP treatment samples compared with the abundance of LAB in the LD and MD groups. In turn, a higher abundance of LAB in the CE and CE+LP treatment samples was detected in the MD group. These results further indicated that CE in the MD group degraded more cellulose and had a greater contribution to the growth of LAB. As expected, high levels of moisture encouraged the growth of *Clostridium* in silage, and the relative abundance of *Clostridium_sensu_stricto_12* in the HD-CK treatment sample reached 36.3%. *Clostridium* is among the typical destructive microorganisms in silage, especially in silage with a high moisture content, and can produce large amounts of butyric acid (Wang et al., 2020), as seen in Table 2.

**FIGURE 4:**
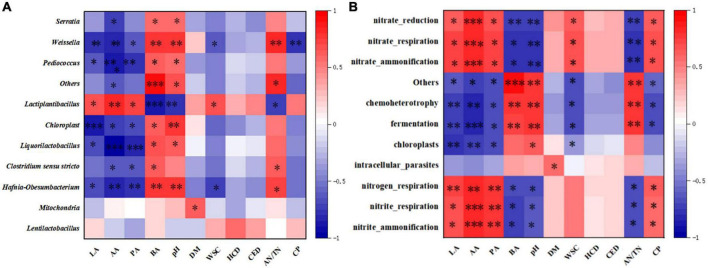
Heatmap of the Spearman correlation coefficients between dominant bacterial species **(A)**/ predicted functions **(B)** and silage fermentation parameters on day 60. The correlations that are positive (closer to 1) have red squares, whereas those that are negative (closer to –1) have blue squares between the taxa and production parameters. Heatmap of the Spearman correlations between function prediction and silage fermentation parameters on day 60. Correlations that are positive (closer to 1) have red squares, whereas those that are negative (closer to –1) have blue squares between the taxa and production parameters. LA, lactic acid; AA, acetic acid; PA, propionic acid; BA, butyric acid; DM, dry matter; WSC, water-soluble carbohydrates; HCD, hemicellulose degradation rate; CED, cellulose degradation rate; AN/TN, ammonia nitrogen/total nitrogen; CP, crude protein; “*,” “^**^,” and “^***^” represent *p* < 0.05, *p* < 0.01, and *p* < 0.001, respectively.

The heat map assembled based on Spearman’s correlation coefficients (Figure 4A) revealed associations between the microbial community and fermentation properties of oat silages. The pH was negatively correlated with the abundance of *Lactiplantibacillus* (*P* < 0.05), consistent with the research result reported by Mu et al. (2020), and was mainly attributed to the metabolites of LAB that could rapidly acidize the silage. It is well known that rapid acidification provides a critical effect in inhibiting unfavorable microbial activity and preserving nutrients (Lv et al., 2020). As expected, we found that *Lactiplantibacillus* in this study was negatively correlated with NH_3_-N concents and positively correlated with CP content, whereas the opposite was observed for *Clostridium_sensu_stricto_12*, which demonstrates the important role of LAB in inhibiting protein breakdown. Moreover, a weaker association between *Lactiplantibacillus* and hemicellulose or cellulose degradation was observed, suggesting that the degradation of fiber is largely contributed by CE. Similar to the CP content, the abundance of *Lactiplantibacillus* was positively correlated with the WSC concents, and *Lactiplantibacillus* showed a positive effect on WSC retention in this study. Compared to *Lactobacillus*, the other LAB (e.g., *Weissella*) may not be as acid-tolerant (Dong et al., 2020), which may explain why *Weissella* was negatively correlated with LA contents and positively correlated with pH, as shown in Figure 4A.

The relationships between the ensiling characteristics and functional predictions of the bacterial community are shown in Figure 4B. As expected, we found that the main organic acid content (LA, AA) in silage was negatively correlated with fermentation and chemical heterotrophic characteristics, while the pH was positively correlated. In addition, as determined in previously published studies (Li P. et al., 2022; Li Y. et al., 2022), AN/TN was negatively related for fermentation and chemoheterotrophy, whereas it was positively related for nitrogen respiration, nitrite respiration and nitrite ammonification, which indicates that protein degradation was inhibited and more proteins were retained. This can also be confirmed by the positive correlation of CP with nitrogen respiration, nitrite respiration and nitrite ammonification.

## Conclusion

As shown in the present study, the inclusion of LAB and CE effectively increased the lactic acid and CP contents in oat silage. A higher degradation rate of lignocellulose and a higher non-structural carbohydrate content were observed after the CE and CE+LP treatment of the MD groups, and provided more fermentation substrates for microorganism. The addition of LP inhibited the growth of unfavorable bacteria, increased the relative abundances of *Lactiplantibacillus*, reduced the microbial diversity and improved the bacterial community structure and function. The combination of LP and CE is more effective in improving the chemical characteristics and fermentation profile than using LP and CE alone, especially for MD group. Taken together, the fermentation effect of ensiling at 60 days was better than that at 30 days. Based on our results, the incorporation of both CE and LP at the moisture content of MD groups ensiled for 60 days is recommended when oats are ensiled. Furthermore, the actions of bacteria and enzymes vary with the moisture content. Further in-depth research on the mechanism underlying the effect of the interaction between the moisture content and bacterial-enzyme actions should be explored, and the results might be helpful to better improve the efficiency of bacterial and enzyme actions in ensiling.

## Data availability statement

The original contributions presented in this study are included in the article/supplementary material, further inquiries can be directed to the corresponding author.

## Author contributions

JX and YZ designed the study and wrote the manuscript. JX, KZ, YL, and XW performed the experiments. ML, QY, HS, QC, YX, CW, PL, CC, and FY conducted the statistical and bioinformatics analyses. YZ was involved in revising the manuscript. All the authors reviewed and approved the final manuscript.
